# Differentiation of Equine Mesenchymal Stromal Cells into Cells of Neural Lineage: Potential for Clinical Applications

**DOI:** 10.1155/2014/891518

**Published:** 2014-11-24

**Authors:** Claudia Cruz Villagrán, Lisa Amelse, Nancy Neilsen, John Dunlap, Madhu Dhar

**Affiliations:** ^1^Department of Comparative and Experimental Medicine, University of Tennessee, Knoxville, TN 37996, USA; ^2^Department of Large Animal Clinical Sciences, University of Tennessee, Knoxville, TN 37996, USA; ^3^Department of Biomedical and Diagnostic Sciences, University of Tennessee, Knoxville, TN 37996, USA; ^4^Advanced Microscopy and Imaging Center, University of Tennessee, Knoxville, TN 37996, USA

## Abstract

Mesenchymal stromal cells (MSCs) are able to differentiate into extramesodermal lineages, including neurons. Positive outcomes were obtained after transplantation of neurally induced MSCs in laboratory animals after nerve injury, but this is unknown in horses. Our objectives were to test the ability of equine MSCs to differentiate into cells of neural lineage *in vitro,* to assess differences in morphology and lineage-specific protein expression, and to investigate if horse age and cell passage number affected the ability to achieve differentiation. Bone marrow-derived MSCs were obtained from young and adult horses. Following demonstration of stemness, MSCs were neurally induced and microscopically assessed at different time points. Results showed that commercially available nitrogen-coated tissue culture plates supported proliferation and differentiation. Morphological changes were immediate and all the cells displayed a neural crest-like cell phenotype. Expression of neural progenitor proteins, was assessed via western blot or immunofluorescence. In our study, MSCs generated from young and middle-aged horses did not show differences in their ability to undergo differentiation. The effect of cell passage number, however, is inconsistent and further experiments are needed. Ongoing work is aimed at transdifferentiating these cells into Schwann cells for transplantation into a peripheral nerve injury model in horses.

## 1. Introduction

Spinal cord and peripheral nerve injuries in horses occur after trauma, toxic/metabolic, and infectious diseases. Less frequently, degenerative and hereditary diseases also pose a threat. These events trigger an inflammatory cascade of events that results in poor performance, disability, or death. Additionally, the impact of peripheral nerve injuries in horses is reflected by big financial and emotional investments. Peripheral nerves can be injured by thermal or chemical injuries, compression, crushing, stretching, or transection [1, 2]. After injury, the blood-nerve barrier permeability increases and an inflammatory response is initiated resulting in proliferation of Schwann cells and activation of local macrophages that respond to tissue damage [2, 3]. Several of these cells secrete neurotrophic factors and other substances that eventually enhance axonal regrowth and remyelination, depending on the magnitude of the lesion and the chronicity of it [1, 2, 4–10]. A loss of axonal continuity along with external nerve structures (neurotmesis, in the Seddon classification) has the poorest prognosis for recovery [1].

Mesenchymal stromal cells (MSCs) from bone marrow and adipose tissue have been demonstrated to transdifferentiate into cells of other lineages other than mesodermal lineages.* In vitro* research has shown the ability of rodent and human MSCs to acquire a neural crest-like cell phenotype after induction with specific culture medium [7–9, 11–13]. Neural protein markers, mainly, vimentin, nestin, *β*_3_ tubulin, and glial fibrillary acidic protein (GFAP), were expressed in these neurally induced cells, although results from different studies are contradictory and hence inconclusive [10, 13–18]. Interestingly, a novel study involving the evaluation of K^+^ and Na^+^ currents in neural crest-like cells revealed that after chemical induction of rat MSCs, these cells appeared to have lost the aforementioned electrophysiological property when compared to noninduced MSCs; concluding that despite morphological and molecular changes similar to neural cells, these chemically induced cells lacked the functional properties of neurons [14]. Nevertheless, several studies have shown positive correlation between* in vitro* and* in vivo* results [2, 7, 9, 12, 14, 19–21]. For instance, bone marrow and adipose-derived undifferentiated MSCs have revealed positive outcomes when these cells were allogeneically transplanted in neurologically injured rats, rabbits, and dogs [2, 9–11, 18–20, 22, 23]. On the other hand, studies in rats have also shown beneficial responses and regain of nerve function after transplanting either chemically induced MSCs into a Schwann cell-like phenotype or pure Schwann cells obtained from fresh peripheral nerve tissue [9, 11, 23, 24]. It is important to mention, however, that in these studies cell therapy occurred immediately after nerve or spinal cord injury.

To our knowledge, there are no reports in the literature that describe the ability of equine bone marrow-derived MSCs (eBM-MSCs) to differentiate into cells of neural lineage or demonstrate the clinical benefits after transplantation into horses suffering from neuropathies. We have previously reported that, to improve the clinical outcomes related to stem cell therapies, it is important to assess the biology and function of MSCs prior to their application in clinical cases [25]. In view of our long term goal of using equine MSCs in neuropathies, the present study was designed as a first step to evaluate the proliferation and survival of eBM-MSCs* in vitro* and their ability to differentiate into neural crest-like cells after chemical induction. We compared these properties and assessed changes, if any, in horses (donors) of two age groups. Additionally, we compared changes in neural differentiation relative to the passage number of eBM-MSC cultures. We used the expression profiles of the neural progenitor markers nestin, vimentin, *β*_3_ tubulin, and GFAP to investigate these changes.

## 2. Materials and Methods

### 2.1. Animals and Bone Marrow Aspiration

Bone marrow aspirates were obtained from the sternum of 2 young mixed breed (range: 1–4 years old) and 4 adult American Quarter Horse (range: 9–13 years old) mares and 1 young American Quarter Horse gelding as described previously [26]. All procedures were carried out as per an approved protocol by the Institutional Animal Care and Use Committee of the University of Tennessee, Knoxville, TN. Briefly, horses were sedated with 0.01-0.02 mg/kg of detomidine hydrochloride, intravenously. A 10 cm band was clipped and surgically prepared on the ventral aspect of the sternal area. Three mL of 2% lidocaine hydrochloride were injected subcutaneously and intramuscularly at the level of the 5th or 6th sternebrae at the ventral midline, followed by a stab incision with a 15-blade on the skin, subcutaneous tissue, and musculature. A Jamshidi needle was inserted perpendicular to the skin until it reached the periosteum. The needle was then forced into the sternum and bone marrow from either the 5th or the 6th sternebrae was obtained by gentle aspiration with a syringe loaded with 1000 IU/10 mL of heparin sulfate.

### 2.2. Isolation and Expansion of MSCs

These procedures were as described earlier [25–27]. Briefly, bone marrow aspirate was diluted with 1X PBS and layered on top of the lymphocyte separation media, Ficoll. After centrifugation at 200 g for 20 min at room temperature, the buffy coat containing the mononuclear cells (MNCs) was obtained. The MNCs were resuspended in growth media containing Dulbecco's Modified Eagle Medium/Ham's F-12 [(DMEM-F12), Cellgro, Manassas, Virginia], 10% fetal bovine serum (FBS), and 1% penicillin/streptomycin. Roughly 20 × 10^6^ cells were seeded in 175 cm^2^ vented tissue culture flasks (Thermo Scientific, Rochester, NY) and maintained at 37°C and 5% CO_2_. First growth medium change was carried out between 5 and 7 days when clusters of colonies were visible under the phase contrast microscope. When 70–80% confluency was reached, the cells were harvested with 0.25% trypsin-EDTA for 2 min at 37°C (passage 1: P1). After centrifugation, the cell pellet was reconstituted either with the growth medium to start a new experiment or with freezing media containing 50% DMEM-F12, 50% FBS, and 5% DMSO for cryopreservation under liquid nitrogen for future experiments. These steps were repeated as needed in order to obtain cells from passage 2 to passage 4 (P2 to P4; low passage) and cells from P9 to P12 (high passage) and used in future experiments.

### 2.3. Demonstration of Stemness on Low Passage Equine MSCs from 7 Donors

#### 2.3.1. Colony Forming Unit Assay (CFU)

Passage 1 of equine MSCs was seeded at a density of 1 × 10^6^ in 100 mm tissue culture dishes (Thermo Scientific, Rochester, NY) and maintained in growth medium for 7 to 10 days until clusters of colonies were observed. The medium was replaced after every 2-3 days. Colonies were fixed with 4% paraformaldehyde and stained with 0.5% of crystal violet (Sigma-Aldrich, Saint Louis, MO). After staining, the dishes were allowed to air-dry and images were acquired with a Fujifilm LAS-4000 imaging system (GE Healthcare Life Sciences).

#### 2.3.2. MTS Proliferation Assay

Low passage (P2–P4) equine MSCs were seeded at a density of 2 × 10^4^ per well in a 24-well tissue culture plate. The medium was replaced every 2-3 days. Cell proliferation was measured at 2, 4, and 7 days after seeding. A CellTiter 96 Aqueous nonradioactive (MTS) assay (Promega, Madison, WI) was used following the manufacturer instructions. Briefly, MTS reagent in a 5 : 1 ratio relative to the media was added to each well and incubated for 3 h at 37°C and 5% CO_2_. The absorbance at 490 nm was measured and data was obtained using Gen5 data analysis software (Biotek, Winooski, Vermont). Growth medium only with no cells was used as a blank to correct the readings for each of the samples.

#### 2.3.3. Induction of Adipogenic, Osteogenic, and Chondrogenic Differentiation

Low passage (P2–P4) of equine MSCs was seeded at a cell density of 2 × 10^5^ cells in 60 mm tissue culture dishes (BD Falcon, New Jersey) and maintained at 37°C and 5% CO_2_ in growth medium. When the cells were roughly 70% confluent, the medium was removed and was replaced with lineage-specific differentiation media as described earlier [27]. Briefly, adipogenic differentiation was induced by the addition of DMEM-F12 medium containing 15% rabbit serum, 1 *μ*mol/L dexamethasone, 10 *μ*g/mL recombinant human insulin, 20 *μ*mol/L indomethacin, and 0.5 mmol/L 3-isobutyl-1-metylxanthine. Osteogenic differentiation was induced by the addition of DMEM-F12 medium containing 100 nmol/L/mL of dexamethasone, 0.25 mmol/L ascorbic acid, and 10 mmol/L *β*-glycerophosphate. Chondrogenic differentiation was induced by the addition of DMEM-F12, 100 nmol/L of dexamethasone, 0.25 mmol/L ascorbic acid, and 5 ng/mL transforming growth factor *β*_1_. Medium was replenished every 2-3 days and differentiation was monitored via microscopic evaluation. Undifferentiated MSCs maintained in regular growth medium without any differentiation reagents, for the same number of days, served as controls. Differentiation was confirmed using lineage-specific cell staining using previously published methods [27]. Specifically, adipogenic cells were stained with oil red-o, osteogenic cells were stained with alizarin red, and chondrogenic cells were stained with alcian blue. Images were acquired with an electronic camera (Nikon DS-Fi2, Japan) connected to a Zeiss microscope and evaluated with the NIS-Elements imaging software (Nikon).

### 2.4. Neural Crest-Like Cell Differentiation on Low and High Passage Equine MSCs from Selected Donors

Neural crest-like cell differentiation was conducted in MSCs from the 7 donors aforementioned. Since we need high numbers of eBM-MSCs for neural differentiation, to obtain appropriate protein samples, we selected one young and one middle-aged donor to conduct the western blot and immunofluorescence experiments. The donors were chosen based on their rates of proliferation as demonstrated by the MTS assay described above.

#### 2.4.1. Cell Culture

Low (P2) and high (P9) of equine MSCs were seeded at a cell density of 8 to 10 × 10^6^ into either polystyrene or Primaria nitrogen-coated 100 mm tissue culture dishes (Becton Dickinson Labware, Bedford, MA). Cells were maintained in regular growth medium at 37°C and 5% CO_2_, for at least 48 h to allow attachment. Neural differentiation was induced using a combination of previously described methods [13, 18]. Briefly, the growth medium was removed and cells were preincubated with medium containing DMEM-F12, 20% FBS, and 1 mM *β*-mercaptoethanol (Sigma-Aldrich) at 37°C and 5% CO_2_, for 18–24 h. Subsequently, the cells were induced by the addition of the neural medium containing DMEM-F12, 2% DMSO, and 200 *μ*M butylated hydroxyanisole (BHA; Sigma-Aldrich) and cells were incubated at 37°C and 5% CO_2_, for 3, 6, 12, 24, or 48 h. Undifferentiated MSCs maintained in regular growth medium for the same number of hours were used as corresponding controls. Undifferentiated and differentiated cells were assayed at the end of each experiment as described below.

#### 2.4.2. Nuclear/Cytoplasmic Staining

Nuclear/cytoplasmic fluorescent staining was used to show the neural cell like morphology of MSCs after neural differentiation. Low and high passages of equine MSCs were seeded at a density of 1.25 × 10^6^ on Primaria nitrogen-coated 60 mm tissue culture dishes and maintained in regular growth medium at 37°C and 5% CO_2_, for at least 48 h to allow attachment. When cells were 80–90% confluent they were chemically induced for neural differentiation as described above. Undifferentiated control MSCs described above were maintained with regular growth medium. For cytoplasmic staining, neurally induced and undifferentiated MSCs at 12 h were stained with 5 *μ*g of WGA (wheat germ agglutinin, Alexa Fluor 488 conjugate; Life Technologies) for 10 min, at room temperature. To stain the nucleus, cells were further washed and stained with 5 *μ*g of TO-PRO-3 iodide stain (Life Technologies, Grand Island, NY) for 10 min, at room temperature. After washing, the cells were mounted with SlowFade Gold Antifade Reagent (Molecular Probes, Grand Island, NY) and images were obtained with a laser scanning spectral confocal microscope (Leica TCS SP2; Leica Microsystems©, Wetzlar, Germany), at 20x and 63x magnification.

#### 2.4.3. Protein Extraction and Western Blot

Total cell lysates were prepared from undifferentiated and neurally induced equine MSCs from low and high passages 12 h after differentiation using standard protocols. Cells on each dish were gently washed with HBSS buffer and collected via cell scraping. To obtain total proteins in each sample, cells were lysed in 200 *μ*L of RIPA buffer (Boston Bioproducts, Ashland, MA) and sonicated and supernatants were obtained by centrifugation. Total protein in each sample was quantitated and concentrations were obtained using modified BCA assay at 660 nm (Pierce, Thermo Scientific). Equal concentrations of total proteins from neurally induced and undifferentiated MSCs were electrophoretically separated in a 10% acrylamide gel and transferred onto nitrocellulose membranes. The membranes were blocked with 5% bovine serum albumin (BSA) and incubated with mouse anti-*β*_3_ tubulin (1 : 1000; Santa Cruz) and mouse anti-GFAP (5 *μ*g/10 mL; 1 : 1000; BD Pharmingen). HRP goat anti-mouse IgG (1 : 5000; BD Pharmingen) was used as the secondary antibody. Antigen detection was performed after exposure to ECL-2 reagent (Pierce, Thermo Scientific). Beta-actin was used as a loading control.

#### 2.4.4. Immunofluorescence (IF)

Low and high passages of equine MSCs were seeded at a density of 1.25 × 10^6^ on Primaria nitrogen-coated 60 mm tissue culture dishes and maintained in growth medium at 37°C and 5% CO_2_, for at least 48 h, to allow attachment. When cells were 80–90% confluent, they were chemically induced for neural differentiation as described above. Undifferentiated MSCs used as controls were maintained with regular growth medium. Neurally induced and undifferentiated MSCs were fixed with 4% paraformaldehyde, permeabilized with 0.1% Triton X-100 (Sigma) for 10 min, at room temperature, and blocked with 5% normal serum for 30 min, at room temperature. Cells were washed and incubated overnight with 5 *μ*g/sample of primary antibodies against nestin (BD Pharmingen) and vimentin (BD Pharmingen), at 4°C. After washing with HBSS buffer, cells were incubated with the secondary antibody (Alexa Fluor 647 donkey anti-mouse IgG at 5 *μ*g per sample; BD Pharmingen) for 20 min, at room temperature. The cells were mounted with SlowFade Gold Antifade with DAPI reagent (Molecular Probes) and images were obtained with a laser scanning spectral confocal microscope (Leica TCS SP2; Leica Microsystems©, Wetzlar, Germany).

### 2.5. Statistical Analysis

A two-tailed Student's* t*-test was used to compare the average of neural crest-like cells that adhered to the polystyrene-coated with those to the Primaria nitrogen-coated tissue culture plates. Similarly, the expression of nestin and vimentin between cells of P2 and P9 from middle-aged horse and between cells of P2 from young and middle-aged horse was compared from the IF data. The expressions of the two proteins were also compared between undifferentiated and neurally induced MSCs. Differences were considered statistically significant when *P* < 0.05.

## 3. Results

### 3.1. Stemness of Equine MSCs

The stemness of cells from all equine MSC cultures was assessed solely in low passage cells. Once the properties of stem cell were demonstrated, only 1 donor from each group (young and middle-aged) was selected and the population of MSCs was passaged for neural experiments.

#### 3.1.1. Morphology

Equine MSC cultures expanded from the bone marrow harvest adhered to the polystyrene surface and displayed spindle-shaped fibroblastic morphology. No morphological change was observed in eBM-MSC cultures generated from young and middle-aged donors.

#### 3.1.2. Proliferation

**Figure 1:**
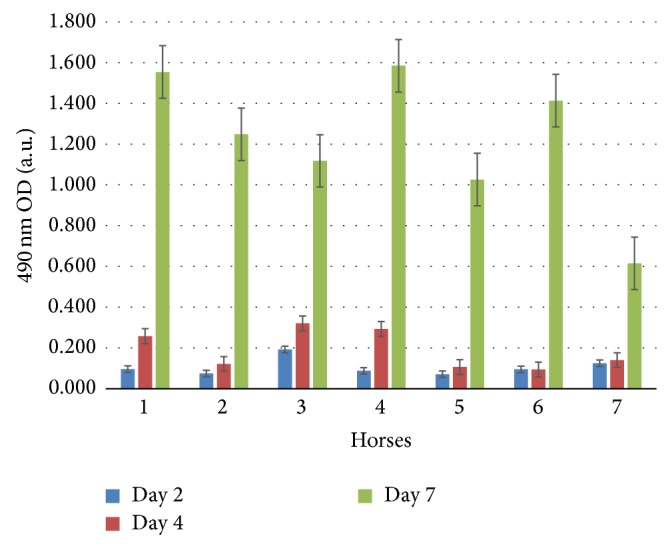
Measurement of rate of proliferation of eBM-MSCs. MTS assay was used to compare the rates of proliferation of eBM-MSCs from 4 young horses (numbers 1–4) and 3 middle-aged horses (numbers 5–7) over a period of 7 days. Absorbance is the optical density (OD) measured at 490 nm with this colorimetric assay. Note the rapid rate of self-renewal on all animals at day 7. Mean values of each sample (*n* = 7) are presented with the standard deviation plotted as the error bars.

Equine MSCs from young (Figure 1: horses 1–4) and middle-aged donors (Figure 1: horses 5–7) were capable of proliferation and viability, which were measured at days 2, 4, and 7. As demonstrated in the figure, there was a 3- to 6-fold increase in absorbance for all the donors over a period of 7 days confirming their proliferation and viability. As expected and previously reported, cellular proliferation was variable between each horse. Horses that showed the highest proliferation and thus generated enough numbers of MSCs in a given period of time were selected for all neural experiments (donors 2 and 6).

#### 3.1.3. CFU

**Figure 2:**
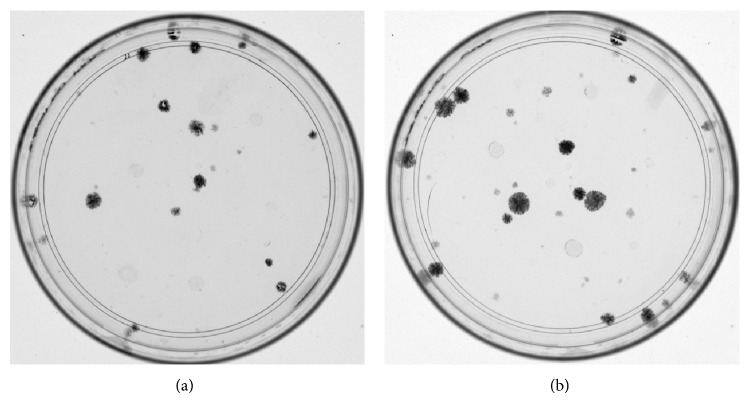
Colony forming unit assay of eBM-MSCs. Representative image of CFU formed after 10 days of seeding passage 1 eBM-MSCs from one young (a) and one middle-aged (b) horse. Note the ability of undifferentiated MSCs from both age groups to form CFU.

Equine MSCs of P1of young and middle-aged horses were capable of growing in clusters over the polystyrene-coated tissue culture dishes at day 10, suggesting that the eBM-MSC cultures established represent the MSCs or the progenitor cells. A representative CFU assay is shown (Figure 2).

#### 3.1.4. Mesodermal Trilineage Differentiation

**Figure 3:**
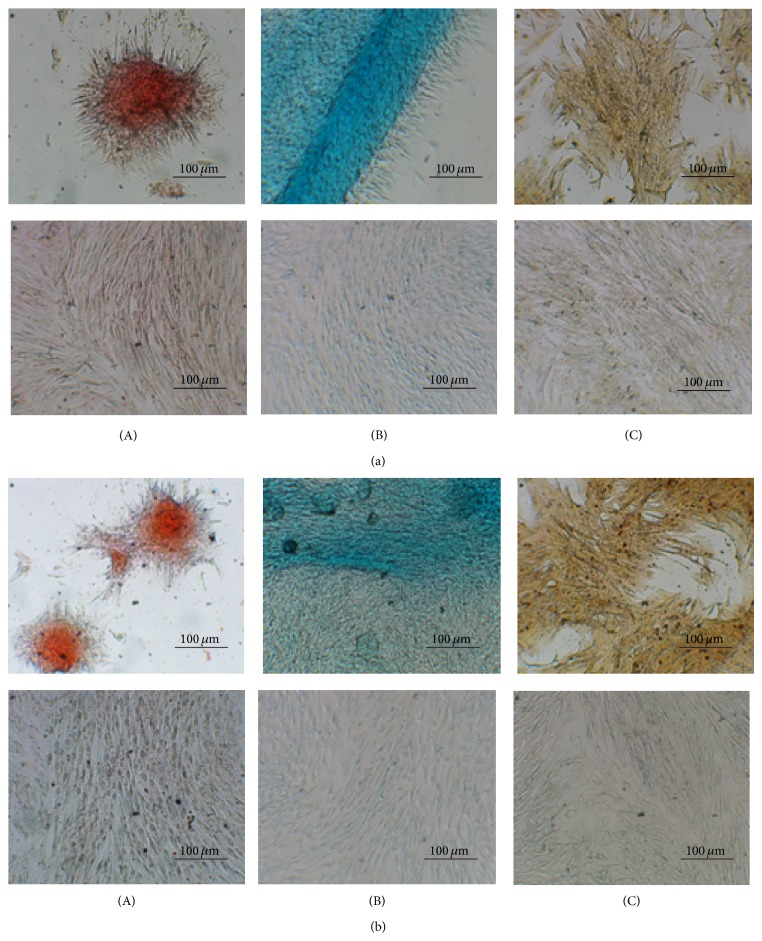
Mesodermal trilineage differentiation assays of eBM-MSCs. Representative images from one young (a) and one middle-aged (b) horse showing oil red-o (A), alcian blue (B), and alizarin red (C) staining of adipogenesis, chondrogenesis, and osteogenesis, respectively, after* in vitro* differentiation. Each undifferentiated control is shown in (b). Scale bar = 100 *μ*m.

Low passage equine MSCs from both young and middle-aged donors were capable of differentiation into adipogenic, chondrogenic, and osteogenic cells under lineage-specific chemical induction (Figure 3). At day 5, adipogenic cells displayed a typical pattern of lipid droplet formation which could be stained with oil red-o. Osteogenic and chondrogenic differentiation potential was confirmed by alizarin red and alcian blue staining, respectively, after 10–15 days of chemical induction. No morphological differences were observed between horses. Variability existed, however, on the time of reaching a differentiated phenotype, as judged by specific staining. Undifferentiated controls remained with a spindle fibroblastic shape at all times and were not positive for lineage-specific staining. Of importance and in accordance with our previously published paper [25], osteogenic and chondrogenic differentiation of MSCs from one middle-age donor (horse 6) consistently occurred earlier than the rest.

All data presented above confirm that equine MSCs generated from each donor are progenitor cells, and they satisfy the criteria to be classified as the adult mesenchymal stromal/progenitor cells.

### 3.2. Neural Crest-Like Cell Differentiation

After ensuring selection of MSCs from low passage cells from the previous experiments, eBM-MSCs were further expanded, passaged, and tested for neural differentiation.

#### 3.2.1. Neural Crest-Like Cell Morphology

**Figure 4:**
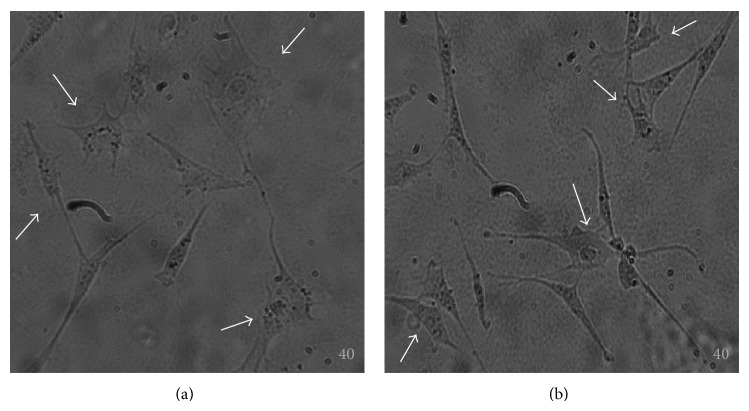
Transdifferentiation assay of eBM-MSCs. Representative images from one young (a) and one middle-aged (b) horse showing neural crest-like cells (white arrows) at 12 h after chemical induction. Note the contraction of the cellular body and the appearance of multiple cell processes. Magnification = 40x.

**Figure 5:**
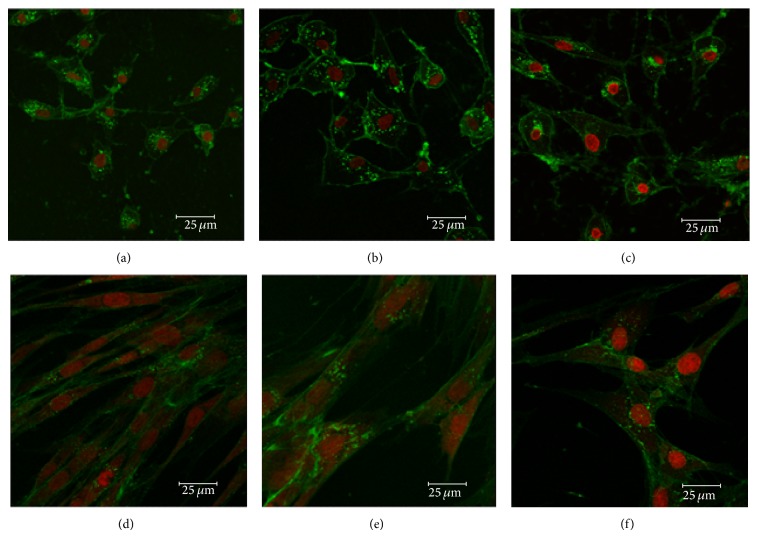
Nuclear/cytoplasmic staining of neural-like eBM-MSCs. Representative confocal images of cytoplasmic (WGA) and nuclear (TO-PRO-3-iodide) fluorescent stains showing the integrity of neural crest-like cells from low passaged eBM-MSCs of one young (a) and one middle-aged (b) horse and from high passaged eBM-MSC of middle-aged (c) horse. Note the typical fibroblast-like morphology of the undifferentiated MSCs (d, e, and f). Scale bar = 25 *μ*m.

For easy visualization, fluorescence microscopy was used to show an intact nucleus and a “healthy” cytoplasm. TO-PRO-3 stain, the most sensitive probe for nucleic acid detection, along with WGA, specific to the cell membrane, was used to demonstrate the nucleus and the cytoplasmic structure of the neural-like cells. Low and high passage eBM-MSCs from the selected young and middle-aged horses were capable of adopting neural crest-like cell morphology as early as 3 h after chemical induction. These morphological characteristics consisted of elongation of the cell, cell body contraction, and formation of one or multiple cell processes (Figure 4). After 12 h of neural differentiation, all the cells displayed neural crest-like cell morphology. Moreover, these cells appeared to grow in a cluster in a mesh-like pattern. Undifferentiated controls had the spindle fibroblastic appearance of a MSC. No differences were microscopically observed on the phenotypical characteristics of neural crest-like cells of low passage between young and middle-aged horses. When analyzing high passage cells, subtle morphological differences were detected. The majority of these cells also displayed the aforementioned neural crest-like cells characteristics; however, they seemed to lose their cytoplasmic integrity and some of them appeared rounded and small. Nuclear and cytoplasmic staining helped in visual assessment of the morphological characteristics of these differentiated cells (Figures 5(a) and 5(b)). These morphological differences were more specific to the passage numbers; that is, they can be attributed to the process of cell culture itself, and not to the age of the donor.

**Figure 6:**
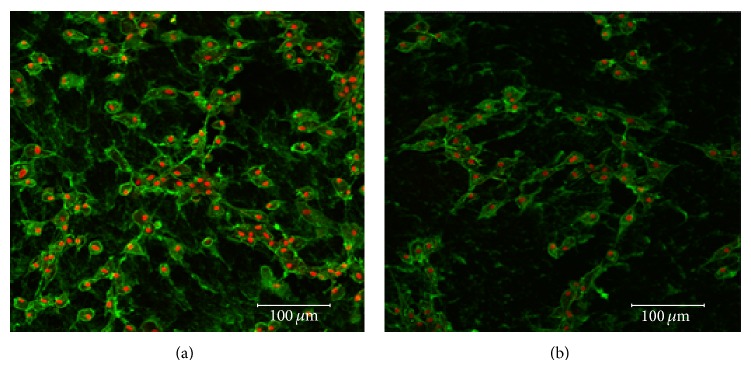
Nuclear/cytoplasmic staining of neural-like eBM-MSCs on different substrates. Representative confocal images of cytoplasmic (WGA) and nuclear (TO-PRO-3-iodide) fluorescent stains showing survival of differentiated cells from one middle-aged horse 12 h after chemical induction, on Primaria, nitrogen-coated (a) and polystyrene-coated (b) tissue culture plates. Note scarce cells on the polystyrene tissue culture plate. Scale bar = 100 *μ*m.

Finally, as judged by the numbers of cells adhered to the 2 different types of tissue culture plates, the nitrogen-coated plates (Primaria) significantly enhanced cell survival and proliferation roughly, 2-fold higher than the polystyrene-coated plates (*P* = 0.0319) after chemical induction (Figure 6).

#### 3.2.2. Expression of Neural Progenitor Proteins

**Figure 7:**
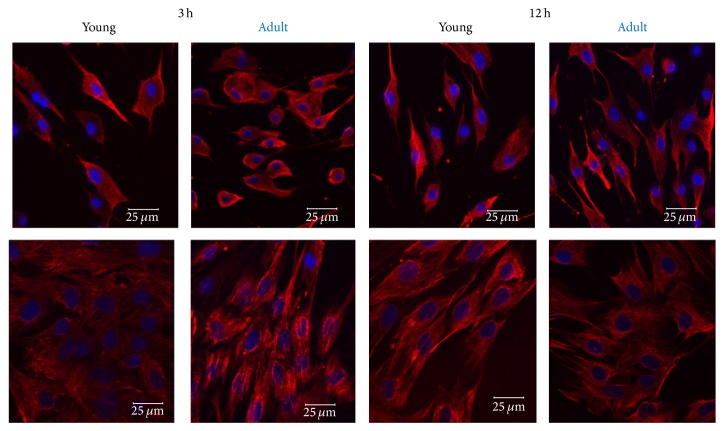
Expression of vimentin in undifferentiated and differentiated low passaged eBM-MSCs. Confocal microscopy shows the expression of vimentin (red) in neural crest-like cells from eBM-MSCs of young and middle-aged horses after 3 h and 12 h of chemical induction. DAPI was used to stain the nuclei (blue). Respective undifferentiated controls are in the lower panel. Note the diffuse localization of vimentin, which is expressed in undifferentiated and in neural progenitor cells. Scale bar = 25 *μ*m.

**Figure 8:**
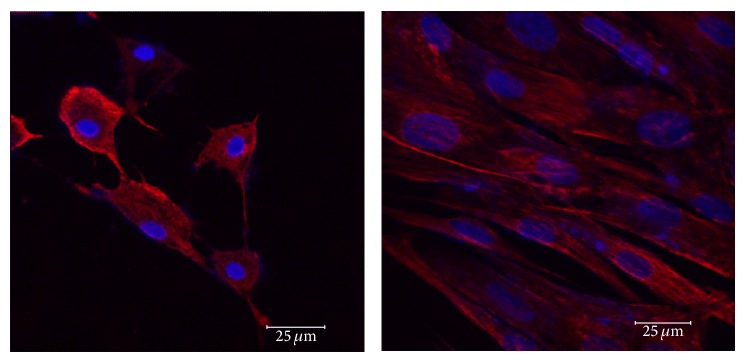
Expression of vimentin in undifferentiated and differentiated high passaged eBM-MSCs. Confocal microscopy shows the expression of vimentin (red) in neural crest-like cells from eBM-MSCs of middle-aged horse after 12 h of chemical induction. DAPI was used to stain the nuclei (blue). Undifferentiated control eBM-MSCs are in the right panel. Note the diffuse localization of vimentin, which is expressed in undifferentiated and in neural progenitor cells. Scale bar = 25 *μ*m.

The expression of vimentin was confirmed by IF (Figures 7 and 8). Vimentin was expressed by cells of low passage for both age groups with no significant differences (*P* = 0.7653); cell passage number did not affect vimentin expression on the middle-age horse (*P* = 0.7774), and no difference on vimentin expression was noted between undifferentiated MSCs and neurally induced MSCs for young (*P* = 0.9660) or middle-aged (*P* = 0.1577) horses.

**Figure 9:**
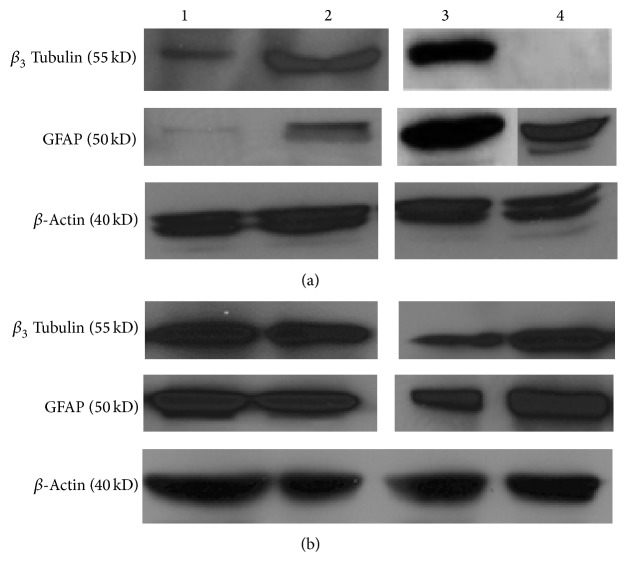
Expression of neural progenitor proteins by immunoblot analysis. Western blot analysis shows the expression of the neural progenitor proteins, *β*_3_ tubulin and GFAP, in differentiated (a) and undifferentiated (b) eBM-MSCs generated from low and high passaged cells from middle-aged (lanes 1 and 2, resp.) and young (lanes 3 and 4, resp.) horses. Beta-actin was used as an internal control. 5 *μ*g of protein was loaded per lane. It is interesting to note the expression of neural progenitor proteins in undifferentiated MSCs, suggesting that the plasticity of equine MSCs may go beyond extramesodermal lineages.

Immunoblot analysis was carried out to assess the expression of GFAP and *β*_3_ tubulin (Figure 9). Differentiated cells from low passaged cells obtained from middle-aged and young MSC cultures showed the expression of both the GFAP and *β*_3_ tubulin (Figure 9(a)). *β*_3_ Tubulin was not expressed in the differentiated cells from high passaged cells obtained from the young MSC culture. The importance, if any, is not known at this time. Interestingly, low and high passaged undifferentiated cells generated from both donors expressed both proteins, suggesting plasticity of MSCs outside of mesodermal lineages (Figure 9(b)).

**Figure 10:**
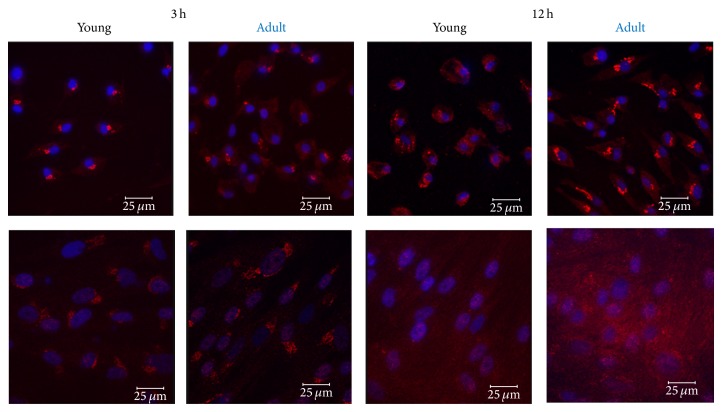
Expression of nestin in undifferentiated and differentiated low passaged eBM-MSCs. Confocal microscopy shows the expression of nestin (red) in neural crest-like cells (upper panels) and in undifferentiated controls (lower panels) from eBM-MSCs of young and middle-aged horses after 3 h and 12 h of chemical induction. DAPI was used to stain the nuclei (blue). Note the predominant perinuclear localization of nestin. Scale bar = 25 *μ*m.

**Figure 11:**
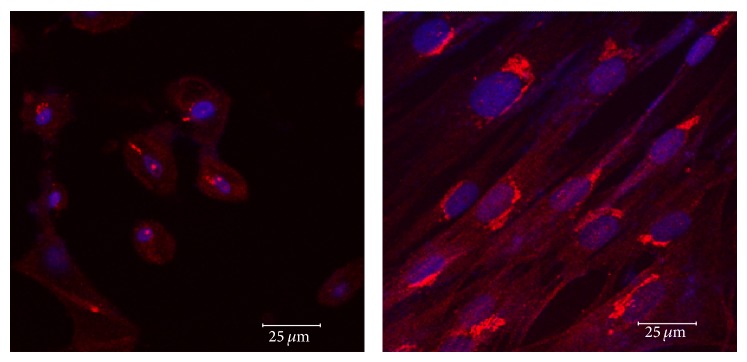
Expression of nestin in undifferentiated and differentiated high passaged eBM-MSCs. Confocal microscopy shows the expression of nestin (red) in neural crest-like cells (left panel) and in undifferentiated controls (right panel) from eBM-MSCs of middle-aged horse after 12 h of chemical induction. DAPI was used to stain the nuclei (blue). Note that nestin is inconsistently found perinuclearly or diffusely in the cytoplasm in the differentiated cells. Expression of nestin in the control is perinuclear. Additionally, based on the intensity of the signal, its expression appears less when compared to cells from the low passage. Scale bar = 25 *μ*m.

We were not able to detect nestin expression by western blot analysis, even when 3 different primary antibodies with various dilutions (1 : 1000, 1 : 2000) were used. Interestingly, nestin expression was evident in undifferentiated and neurally differentiated MSCs of low and high passages for both age groups by IF (Figure 10). Nestin expression on cells of low passage for both age groups was observed with no significant differences (*P* = 0.7325). Additionally, cell passage number did not affect significantly the expression of nestin on the middle-aged horse (*P* = 0.3467), and no difference on nestin expression was noted between undifferentiated MSCs and neurally induced MSCs for young (*P* = 0.9616) or middle-aged (*P* = 0.0830) horses. A perinuclear location of nestin was evident on undifferentiated and neurally differentiated MSCs of low passage and on undifferentiated MSCs of high passage of both age groups (Figure 10). The location of nestin in neurally differentiated cells of high passage, however, was inconsistent, with some cells displaying a perinuclear location and others showing a more diffuse, cytoplasmic expression (Figure 11). It is possible that cells of high passages undergo changes that alter the structure of some filamentous proteins (i.e., nestin).

## 4. Discussion

Peripheral nerve injuries are a cause of poor performance in horses. These injuries are difficult to manage and treatment mostly relies on physical therapy and anti-inflammatories; however, the long-term effects are time and personnel consuming. The development of nervous system cells is divided into various stages. After determination of their fate, these cells migrate to specific locations of the nervous system and accomplish different functions [18, 28]. Previous studies have demonstrated that undifferentiated MSCs are able to express some neural protein markers, leading to the question whether MSCs are in advance committed to a neural lineage [29–32]. Moreover, as neural progenitors develop into more specialized cells, changes in protein markers are also evident [4, 13, 17, 30, 33].

Adipose-derived and bone marrow-derived MSCs from humans and rats are able to differentiate into neural lineages [4, 13, 18, 28, 29, 33–37]. Several reports have described different methods of chemical induction for neural differentiation [13, 30, 33, 37, 38]. Medium containing transretinoic acid or butylated hydroxyanisole is the most frequently reported. Additionally, coculture systems with MSCs and cells from nervous tissue have also proven successful for the induction of neural differentiation of MSCs [29].

We induced trilineage differentiation (osteogenic, chondrogenic, and adipogenic) on bone marrow-derived MSCs from young and middle-aged horses to characterize their plasticity into mesodermal lineages. We also induced these cells to differentiate into cells of neural lineage to assess their plasticity outside of mesodermal lineages. Cells from all horses were capable of proliferation and underwent differentiation into adipogenic, osteogenic, and chondrogenic lineages. This was consistent with a previous report published from our laboratory [25] and, thus, confirmed the stemness of the cultured MSCs. We cannot ensure cell self-renewal based on the capability of proliferation of these cells. We, however, can just hypothesize that equine MSCs may be capable of self-renewal based on the fact that the cells remained under undifferentiated state during the experiment and based on the fact that these cells were also capable of multipotency when induced with different medium conditions. For the neural differentiation, fluorescence microscopy revealed that morphological changes were observed as early as 3 h after chemical induction in all horses. Using nuclear/cytoplasmic staining we were able to demonstrate that differentiated cells acquired a neural crest-like cell morphology in which retraction of the cell soma and formation of multiple cell processes were observed. Most importantly, the proliferation of differentiated MSCs under the neural media was higher on the nitrogen-coated plates (Primaria) than on the polystyrene tissue culture plates, suggesting stabilization of the cell membrane by providing a positive charge.

By the combination of western blot and IF analyses, we found that all MSCs, undifferentiated and differentiated, expressed neural progenitor markers; namely, vimentin, nestin, GFAP, and *β*_3_ tubulin were evident. The expression of GFAP and *β*_3_ tubulin in undifferentiated MSCs was particularly interesting and data are supported by previous studies in rats and humans, suggesting their capability for neural lineage differentiation [39, 40]. Nestin has been shown to be expressed in muscle and neural progenitor cells, as well as in highly proliferative cells (i.e., following injuries, high mitotic rate, etc.) [41–43]. In IF data, nestin subjectively appeared weaker in cells from high passage than cells from low passage, suggesting the loss of cellular mechanisms for proliferation and plasticity as the cell ages. We detected a perinuclear location of nestin in undifferentiated and neural crest-like cells from P2. In differentiated cells from P9 nestin was expressed perinuclearly but, also extended to the cytoplasm. This was similar to previous reports [43, 44] and may suggest posttranslational modifications of nestin [42–44], the analysis of which was beyond the scope of our study. Similarly, the effect of cell passage number on the expression of these markers also suggested modifications or structural changes as the cells age, but further studies are needed to confirm this.

Our study mainly relies on morphological changes and neural marker protein expression to describe the events occurring during neural differentiation of eBM-MSCs. Our results agree with previous* in vitro* studies performed in bone marrow-derived MSCs from rat, human, and dogs in which eBM-MSCs are chemically induced into cells that display morphologic, genetic, and protein characteristics of neural progenitors. To our knowledge, this is the first report describing the plasticity, morphological characteristics, and protein expression changes of eBM-MSCs into cells of neural lineage after chemical induction. We have not only demonstrated the* in vitro* differentiation patterns but also validated the cross-reactivity of human neural-specific antibodies with equine protein samples. Further studies are, however, warranted for investigating the functionality of these horse cells* in vivo*.
